# Effect of Berberine on Cardiovascular Disease Risk Factors: A Mechanistic Randomized Controlled Trial

**DOI:** 10.3390/nu13082550

**Published:** 2021-07-26

**Authors:** Jie V. Zhao, Wai-Fung Yeung, Yap-Hang Chan, Dana Vackova, June Y. Y. Leung, Dennis K. M. Ip, Jiaxi Zhao, Wai-Kwan Ho, Hung-fat Tse, Catherine Mary Schooling

**Affiliations:** 1 School of Public Health, Li Ka Shing Faculty of Medicine, The University of Hong Kong, Hong Kong, China; owen22@hku.hk (W.-F.Y.); vackova@hku.hk (D.V.); leungjy@hku.hk (J.Y.Y.L.); dkmip@hku.hk (D.K.M.I.); cms1@hku.hk (C.M.S.); 2 Division of Cardiology, Queen Mary Hospital, Hong Kong, China; cyh472@ha.org.hk (Y.-H.C.); hftse@hkucc.hku.hk (H.-f.T.); 3 Department of Pharmacology and Pharmacy, The University of Hong Kong, Hong Kong, China; jxzhao@hku.hk; 4 Department of Chinese Medicine, Pok Oi Hospital, Hong Kong, China; cmscp1@pokoi.org.hk; 5 Department of Medicine, Li Ka Shing Faculty of Medicine, The University of Hong Kong, Hong Kong, China; 6 School of Public Health and Health Policy, City University of New York, New York, NY 10027, USA

**Keywords:** berberine, randomized controlled trial, testosterone

## Abstract

Cardiovascular disease (CVD) is a major contributor to the global burden of disease. Berberine, a long-standing, widely used, traditional Chinese medicine, is thought to have beneficial effects on CVD risk factors and in women with polycystic ovary syndrome. The mechanisms and effects, specifically in men, possibly via testosterone, have not been examined previously. To assess the effect of berberine on CVD risk factors and any potential pathway via testosterone in men, we conducted a randomized, double-blind, placebo-controlled, parallel trial in Hong Kong. In total, 84 eligible Chinese men with hyperlipidemia were randomized to berberine (500 mg orally, twice a day) or placebo for 12 weeks. CVD risk factors (lipids, thromboxane A2, blood pressure, body mass index and waist–hip ratio) and testosterone were assessed at baseline, and 8 and 12 weeks after intervention. We compared changes in CVD risk factors and testosterone after 12 weeks of intervention using analysis of variance, and after 8 and 12 weeks using generalized estimating equations (GEE). Of the 84 men randomized, 80 men completed the trial. Men randomized to berberine had larger reductions in total cholesterol (−0.39 mmol/L, 95% confidence interval (CI) −0.70 to −0.08) and high-density lipoprotein cholesterol (−0.07 mmol/L, 95% CI −0.13 to −0.01) after 12 weeks. Considering changes after 8 and 12 weeks together, berberine lowered total cholesterol and possibly low-density lipoprotein-cholesterol (LDL-c), and possibly increased testosterone. Changes in triglycerides, thromboxane A2, blood pressure, body mass index and waist–hip ratio after the intervention did not differ between the berberine and placebo groups. No serious adverse event was reported. Berberine is a promising treatment for lowering cholesterol. Berberine did not lower testosterone but instead may increase testosterone in men, suggesting sex-specific effects of berberine. Exploring other pathways and assessing sex differences would be worthwhile, with relevance to drug repositioning and healthcare.

## 1. Introduction

Cardiovascular disease (CVD) is a major contributor to the global burden of morbidity and mortality, with higher incidence and mortality rates in men than in women [1,2], and a corresponding need for, possibly sex-specific, innovations in prevention and treatment. Recently, development of new therapeutics for CVD prevention and treatment focusing on addressing lipid and inflammatory pathways has encountered repeated expensive failures, which has discouraged investment [3].

Berberine, an isoquinoline plant alkaloid [4], is widely used in traditional Chinese and Ayurvedic medicine [5]. Berberine is thought to have a range of beneficial effects on the cardiovascular system, including anti-lipidemic [5,6] and anti-inflammatory properties [5], with good safety [5,6,7,8]. Berberine is highly concentrated in the roots, rhizomes and stem bark of several plants, such as Coptis (Huanglian), Rhizoma coptidis and Hydrastis canadensis (goldenseal) [9]. Berberine is extracted from Coptis (Huanglian) and Phellodendron Chinese (Huangbai), to make into compound berberine tablets, currently used in clinical practice for digestive diseases [8]. Berberine is also widely used as a nutrient supplement in the US.

Although berberine has been in use for thousands of years, the mechanisms by which berberine exerts its effects are unclear. Several pathways have been proposed. One possibility is activation of the adenosine monophosphate-activated protein kinase (AMPK) pathway that coordinates energy balance and lipid synthesis [10,11]. AMPK is also targeted by an established treatment for diabetes, i.e., metformin. Another possibility is inhibition of the aldo-keto reductase family 1 member C3 (AKR1C3), that regulates steroid hormone action, such as estrone to 17β-estradiol or androstendione to testosterone conversion [12]. Sex hormones have long been thought relevant to CVD because of the difference in incidence by sex. Estrogen has been extensively investigated, but is no longer thought to be highly relevant [13], while the role of androgens is being more extensively investigated [14] for empirical and theoretical reasons [15,16]. A Mendelian randomization study, to minimize confounding, in Chinese men suggested that higher testosterone may be associated with more unfavorable cardiac function [17] and unhealthier lipids, i.e., including lower high-density lipoprotein cholesterol (HDL-c) [18]. A meta-analysis of RCTs suggested that testosterone lowers HDL-c [19], and raises the risk of thrombosis [20], possibly by inducing platelet aggregation via thromboxane A2 [21,22]. Berberine has been reported to lower total testosterone in women [23], whilst effects on testosterone in men have not been examined.

Systematic reviews and meta-analyses of clinical trials have shown a lipid-lowering effect of berberine in people with hyperlipidemia with or without coronary heart disease or diabetes [5,6,24], as well as potential benefits on blood pressure and adiposity [8,25,26], with no serious adverse events [5,6,8,27]. However, these trials vary in quality and design, making meta-analyses difficult to interpret [6]. Publication bias is also possible [24]. To address these gaps and the case for translation, we conducted a mechanistic, parallel, randomized, double-blind, placebo-controlled trial in Chinese men with hyperlipidemia to examine the effects of berberine on a set of well-established CVD risk factors, specifically, lipids, thromboxane A2, systolic and diastolic blood pressure and adiposity. We also assessed the hypothesis that berberine lowers testosterone, and if so, whether it mediates the effect of berberine on CVD risk factors. We limited the study to men because men have a substantially higher rate of CVD than women [2,28].

## 2. Materials and Methods

### 2.1. Study Design

This is a randomized, parallel, double-blind, placebo-controlled trial conducted in Hong Kong. This trial was registered in ClinicalTrials.gov of the United States (NCT03770325) and conducted according to a pre-specified protocol. The trial was conducted following the requirement of International Council for Harmonisation (ICH) Good Clinical Practice (GCP) standards, and reported according to the Consolidated Standards of Reporting Trials (CONSORT) guidelines.

### 2.2. Subjects

We recruited volunteers from staff and/or their families at the University of Hong Kong and from an out-patient clinic in the Department of Medicine, Queen Mary Hospital, with the sample size calculated based on previous studies (as shown in the Supplementary Methods [29,30]), according to the following inclusion criteria: (1) age 20 to 65 years, (2) of Chinese ethnicity, (3) with hyperlipidemia, defined following the National Cholesterol Education Program Adult Treatment Panel III [31] as triglycerides greater than 150 mg/dl (1.70 mmol/L), total cholesterol greater than 200 mg/dl (5.16 mmol/L), and/or LDL-c greater than 100 mg/dl (2.58 mmol/L), (4) willing to make return visits, (5) not having received hormone replacement therapy, such as testosterone replacement therapy, in the past 12 months, (6) not currently taking berberine or nutraceuticals that contain berberine, (7) free of any congenital conditions, including familial hypercholesterolemia, (8) free of any infectious diseases, e.g., seasonal influenza, (9) with no history of chronic diseases including ischemic heart disease, myocardial infarction, stroke, diabetes, cancer and liver or renal dysfunction. The participants were given an assessment report and a supermarket voucher, worth 300 HKD (~38 US dollars), at the end of the study as an incentive.

### 2.3. Ethical Considerations

Ethical approval was obtained from the Institutional Review Board of the University of Hong Kong/Hospital Authority Hong Kong West Cluster (UW18-037). The trial would have been discontinued immediately after receiving notification of any serious clinical adverse event. All participants were provided full information about the trial, informed that participation was voluntary and that they could decline further participation at any time without penalty, and signed a consent form before participation, including consent for use of left-over samples for future research.

### 2.4. Intervention and Study Outcomes

Intervention: purified berberine tablets (500 mg orally twice a day), which have been shown to be safe and effective in lipid-lowering in previous trials [7,32], or placebo tablets, prepared with the same appearance, were administered for 12 weeks.

Primary outcomes: lipids (total cholesterol, low-density lipoprotein cholesterol (LDL-c), triglycerides, HDL-c, thromboxane A2, systolic and diastolic blood pressure, and serum testosterone).

Secondary outcomes: Body mass index (BMI) and waist–hip ratio (WHR).

### 2.5. Randomization, Allocation Concealment and Blinding

Computer-generated random numbers were used to randomize half the participants to berberine, and half to placebo. A statistician prepared identical, opaque envelopes, with a unique identifier comprised of the random number on the cover of each envelope, and prepared berberine or placebo according to the corresponding allocation code. The allocation codes were only known to the statistician who prepared them. Participants and all others involved in the study, including those conducting the follow-up and performing the laboratory tests, did not know the link between the unique identifier and the allocation code.

### 2.6. Data Collection

Participants who agreed to participate were assessed for eligibility at the first visit, according to the inclusion criteria. At the second visit, eligible participants provided informed consent, and were provided a copy of the consent and information on how to contact the study staff at all times. Participants were then randomized to the berberine or placebo group. Participants completed a questionnaire including medical history, smoking status, alcohol use, physical activity, use of lipid-lowering therapies and socio-demographics. Fasting blood (20 mL) was taken for biochemical assessment and the remaining was stored at −80 °C. Weight, height, WHR and blood pressure were measured. An automated oscillometric device was used for blood pressure measurement.

When the participants returned at 8 weeks (third visit) and 12 weeks (fourth visit) post-intervention, the same assessments were performed using the same procedures. Participants were asked whether any discomfort had occurred during the study. After completion of all the visits, participants were given an assessment report and the supermarket voucher.

### 2.7. Biochemical Assessment

Blood samples were tested using a Hitachi automated analyzer for lipids, an enzyme-linked immunosorbent assay (ELISA) for thromboxane A2 and a competitive immunoassay for serum testosterone.

### 2.8. Statistical Analysis

Intention to treat analysis according to the randomization is the principal of analysis, where applicable. In the situation of loss to follow-up, as recommended by the Cochrane Handbook [33], an available case analysis was used. We calculated changes after 8 and 12 weeks and assessed differences between the berberine and placebo groups using analysis of variance. We assessed whether changes in serum testosterone differed between groups after 8 or 12 weeks, and if so, we assessed mediation by testosterone. We also used a generalized estimating equation (GEE) model with an exchangeable correlation matrix [34]. The GEE model uses the quasi-likelihood estimation, and is being increasingly applied to analyze longitudinal and other correlated data [35]. Given that we had repeated post-treatment measurements, we used the GEE model to make the best use of all the data. All statistical analyses were conducted using R version 4.0.1 (R Foundation for Statistical Computing, Vienna, Austria).

## 3. Results

**Figure 1:**
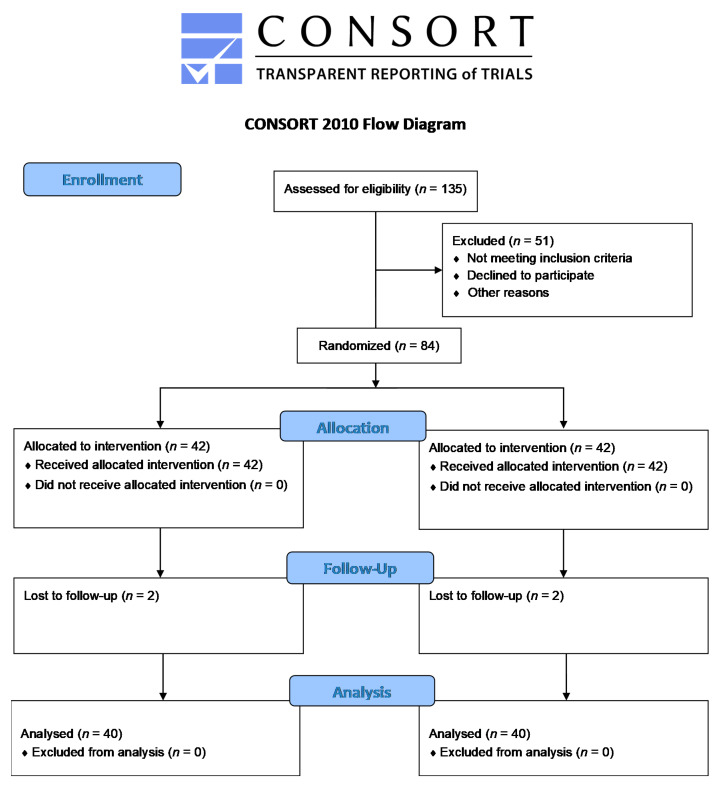
Flow chart.

Using recruitment by advertisements, email and distribution of leaflets, 135 men showed interest in the trial. Of these 135 men interested in the trial, we included 84 eligible men, with 42 in the berberine group and 42 in the placebo group (Figure 1). Two men in each group withdrew due to not feeling well (Supplementary Table S1) and for other personal reasons, leaving 40 men in each group for analysis. Characteristics were similar in both groups (Table 1).

After 12 weeks of intervention, men taking berberine had a larger reduction in total cholesterol and HDL-c than those taking placebo (Table 2). Similar effects on total cholesterol were evident after 8 weeks of intervention (Table 3). Consistent with previous studies [5,27], berberine was safe with no serious adverse events (Supplementary Table S1).

Using a GEE model to include information at both timepoints (8 and 12 weeks) obtained a similar estimate for total cholesterol and no association for HDL-cholesterol (Table 4). However, the reduction in LDL-c and the increase in testosterone were statistically significant (Table 4). No differences in triglycerides, thromboxane A2, blood pressure, BMI or WHR were evident.

## 4. Discussion

Our findings extend the current evidence by assessing the effect of berberine in men only, including the effect on testosterone. This RCT in men suggests that berberine affects CVD risk factors by lowering total cholesterol, as well as possibly lowering LDL-c and increasing testosterone.

Our findings are consistent with previous studies showing a beneficial effect of berberine on total cholesterol and possibly LDL-c [5,6,24]. The reduction in HDL-c might have occurred by chance at 12 weeks after treatment because it was not evident when using information at both 8 and 12 weeks, which needs replication. We found a null effect of berberine on thromboxane A2, the effect of which has not been examined previously. Differences in triglycerides, blood pressure and adiposity between the groups were not statistically significant, which is less consistent with some systematic reviews and meta-analyses of RCTs [8,24,25], although the direction of effects were consistent. Moreover, high heterogeneity in the RCTs included in previous meta-analyses, with I^2^ up to over 90% [6,24], make their results inconclusive and difficult to interpret. For example, the included trials have varying dosages (from 0.9 to 1.5 g daily), intervention periods (from 1 month to 2 years) and quality, and few studies were publicly registered [6]. A previous systematic review and meta-analysis also showed that berberine did not lower BMI, but lowered WHR in women [26]. It is also possible that our study was conducted in men without established coronary heart disease or diabetes, whilst berberine may have more obvious effects on CVD risk factors in people with CVD and/or diabetes. As such, it would be worthwhile to replicate an RCT using a larger sample size and assess a wider range of lipids, particularly apolipoprotein B (apoB), which is increasingly thought to be the key lipid relevant to higher risk of CVD [36,37].

To our knowledge, our study has for the first time examined the effect of berberine on testosterone in men, whilst previous RCTs of berberine on sex hormones generally focused on women with polycystic ovary syndrome (PCOS) [23,38]. Supplementation with berberine (1500 mg/day for 3 months) lowered testosterone in women with PCOS in two similar sized trials (50 women in each group in one trial [23], and 33 versus 36 women in another trial [38]). In contrast, our study suggests that berberine has different effects on testosterone in men than those found in women. However, the previous trials in women were conducted in women with PCOS [23,38], who tend to have higher testosterone, and effects might be different in women with normal endocrine parameters. As such, to assess any sex difference in the endocrine effects of berberine, an RCT in women without PCOS is needed. Similar sex-specific effects on testosterone have also been suggested for metformin [39], the first-line treatment for type 2 diabetes, which like berberine targets AMPK. Given the similarity and the beneficial effect of testosterone on glucose metabolism [40,41], further examination of the effects of berberine on glucose and endocrine factors, including factors relevant to testosterone, such as sex hormone binding globulin, might also be worthwhile. Considering that sex differences in response to drugs are increasingly receiving attention [42], it would be worthwhile to conduct another trial in women in the same setting for comparison, to identify and clarify differential effects of berberine in men and women.

Our study has several strengths. First, our study followed a pre-specified protocol, which avoids selective reporting. Second, our study examined the effect of berberine on testosterone and thromboxane A2 in men for the first time, which may also serve as a reference for future studies on sex-specific effects of berberine. Nevertheless, limitations of the study exist. First, we recruited the participants from the University of Hong Kong and Queen Mary Hospital to facilitate recruitment, and explained to potential recruits the purpose of the study, which might affect participation. However, the randomization removes confounding. The outcomes are also objective measures from a blood test which are unaffected by these factors. Second, the sample size of this study was relatively small. The sample size was estimated based on previous studies [32,43]. Power might be insufficient if the effect size is lower than previously reported, given the heterogeneity in different studies [6]. We also used the GEE model to make the best use of the data. Third, testosterone varies by time of day, however, we collected all the samples in the morning. Fourth, loss to follow-up lowers the sample size and thus lowers the precision in the estimates. However, the directions of estimates are unchanged. Fifth, thromboxane A2 has high variability, so we cannot exclude the possibility that the null effect is due to a lack of power. Sixth, findings in Chinese might not apply to other populations, such as Europeans of different ethnicity. However, causal effects are expected to be consistent across settings, although their relevance might vary by setting [44]. As such, the directions of effects are not expected to be different by population.

Our findings, together with previous evidence, suggest that berberine may lower cholesterol, with good safety. In comparison with previous evidence in women [23,38], our study also indicates that berberine might have sex-specific effects on sex hormones. Exploring other underlying pathways and assessing the sex disparity would be worthwhile, with direct relevance to drug repositioning and healthcare.

## 5. Conclusions

Our findings extend the current evidence by assessing the effect of berberine in men only. Our study showed that berberine lowers total cholesterol and possibly LDL-c, with good safety. Our study also adds to the research by assessing the effect of berberine on testosterone and thromboxane A2 in men. Berberine did not lower testosterone in men, but instead possibly increased testosterone, in contrast to berberine reducing testosterone in women. Explicating mechanisms underlying any differences by sex and further exploring other pathways, including a wide range of lipids, glucose metabolism and endocrine factors, would be worthwhile.

## Figures and Tables

**Table 1:** Baseline characteristics of the participants in the berberine and placebo groups.

Characteristics	Number (Percentage) or Mean (SD) *	
	Berberine Group	Placebo Group
Place of birth		
- Hong Kong	36 (90%)	33 (82.5%)
- Macau	0 (0%)	2 (5%)
- Mainland China	4 (10%)	5 (12.5%)
Education level		
- Primary school	6 (15.0%)	7 (17.5%)
- High school	25 (62.5%)	20 (50%)
- University and above	9 (22.5%)	13 (32.5%)
Smoking status		
- Non-smoker	24 (60%)	30 (75%)
- Ex-smoker	12 (30%)	5 (12.5%)
- Current smoker	4 (10%)	5 (12.5%)
Alcohol drinking		
- Never	6 (15.0%)	8 (20%)
- Ex-drinker	10 (25.0%)	8 (20%)
- Less than 1 day per week	15 (37.5%)	15 (37.5%)
- 1–2 days per week	3 (7.5%)	7 (17.5%)
- 3–7 days per week	6 (15%)	2 (5%)
Age (year)	49.5 (11.1)	44.8 (13.5)
Physical activity		
Time of doing vigorous physical activity per day (minutes)	85.0 (43.7)	71.0 (60.9)
Time of doing moderate physical activity per day (minutes)	69.6 (51.0)	76.8 (83.9)
Time of doing light physical activity per day (minutes)	67.3 (61.2)	60.0 (59.9)

SD, standard deviation. * Number (percentage) was used for categorical variables, mean (SD) was used for continuous variables.

**Table 2:** Baseline and 12-week post-treatment outcomes in the berberine and placebo groups.

Outcomes	Berberine (Mean (SD))		Placebo (Mean (SD))		Comparing the Changes in Two Groups (Change (Berberine)–Change (Placebo))		
	Baseline	12-Week Treatment	Baseline	12-Week Treatment	beta	95% CI	*p*
Total cholesterol (mmol/L)	5.4 (0.9)	4.9 (1.0)	5.5 (1.1)	5.4 (1.0)	−0.39	−0.70, −0.08	0.02
LDL-cholesterol (mmol/L)	3.3 (0.8)	3.0 (0.8)	3.5 (1.0)	3.4 (0.9)	−0.21	−0.50, 0.08	0.15
Triglycerides (mmol/L)	1.9 (0.8)	1.6 (0.9)	1.8 (1.3)	1.6 (1.0)	−0.08	−0.37, 0.21	0.60
HDL-cholesterol (mmol/L)	1.17 (0.3)	1.13 (0.3)	1.21 (0.4)	1.24 (0.4)	−0.07	−0.13, −0.01	0.03
SBP (mmHg)	131 (16.8)	130 (17.8)	125 (12.7)	125 (11.6)	−1.53	−7.47, 4.41	0.62
DBP (mmHg)	88.3 (13.8)	86.6 (13.0)	82.1 (10.8)	81.0 (9.0)	−0.58	−4.52, 3.36	0.78
BMI	26.1 (3.8)	26.0 (4.0)	26.3 (3.7)	26.5 (3.7)	−0.27	−0.74, 0.20	0.26
WHR	0.93 (0.06)	0.92 (0.06)	0.91 (0.05)	0.91 (0.06)	−0.01	−0.03, 0.01	0.26
Thromboxane A2 (pg/mL)	188 (109)	255 (162)	198 (107)	243 (138)	21.46	−21.7, 64.6	0.33
Testosterone (nmol/L)	14.3 (4.5)	15.0 (4.6)	15.8 (5.8)	15.1 (5.4)	1.43	−0.10, 2.96	0.07

Abbreviations: LDL: Low Density Lipoprotein; HDL: High Density Lipoprotein; SBP: systolic blood pressure; DBP: diastolic blood pressure; BMI: Body Mass Index; WHR: waist–hip ratio.

**Table 3:** Baseline and 8-week post-treatment outcomes in the berberine and placebo groups.

Outcomes	Berberine (Mean (SD))		Placebo (Mean (SD))		Comparing the Changes in Two Groups (Change (Berberine)–Change (Placebo))		
	Baseline	8-Week Treatment	Baseline	8-Week Treatment	beta	95% CI	*p*
Total cholesterol (mmol/L)	5.4 (0.9)	4.6 (0.9)	5.5 (1.1)	5.1 (1.2)	−0.39	−0.72, −0.06	0.03
LDL-cholesterol (mmol/L)	3.3 (0.8)	2.9 (0.8)	3.5 (1.0)	3.3 (0.9)	−0.25	−0.54, 0.04	0.11
Triglycerides (mmol/L)	1.9 (0.8)	1.5 (0.8)	1.8 (1.3)	1.9 (2.2)	−0.53	−1.22, 0.16	0.13
HDL-cholesterol (mmol/L)	1.17 (0.3)	1.07 (0.3)	1.21 (0.4)	1.10 (0.5)	0.01	−0.09, 0.11	0.86
SBP (mmHg)	131 (16.8)	129 (21.8)	125 (12.7)	124 (10.6)	−0.30	−5.32, 4.72	0.91
DBP (mmHg)	88.3 (13.8)	86.7 (16.7)	82.1 (10.8)	80.5 (12.3)	−0.05	−4.83, 4.73	0.98
BMI	26.1 (3.8)	25.5 (5.3)	26.3 (3.7)	26.2 (3.6)	−0.51	−1.63, 0.61	0.38
WHR	0.93 (0.06)	0.93 (0.06)	0.91 (0.05)	0.91 (0.05)	−0.001	−0.02, 0.02	0.95
Thromboxane A2 (pg/mL)	188 (109)	214 (104)	198 (107)	228 (145)	−4.5	−51.1, 42.1	0.85
Testosterone (nmol/L)	14.3 (4.5)	14.7 (4.7)	15.8 (5.8)	15.0 (5.2)	1.19	−0.18, 2.56	0.09

**Table 4:** The effect of berberine on CVD risk factors and testosterone using a GEE model ^*^.

Outcomes	Beta	95% CI	*p*
Total cholesterol (mmol/L)	−0.39	−0.62, −0.16	0.001
LDL-cholesterol (mmol/L)	−0.23	−0.43, −0.02	0.03
Triglycerides (mmol/L)	−0.31	−0.67, 0.06	0.10
HDL-cholesterol (mmol/L)	−0.03	−0.09, 0.02	0.26
SBP (mmHg)	−0.91	−4.75, 2.93	0.64
DBP (mmHg)	−0.31	−3.42, 2.80	0.84
BMI	−0.39	−0.99, 0.21	0.20
WHR	−0.006	−0.02, 0.01	0.36
Thromboxane A2 (pg/mL)	8.46	−23.0, 39.9	0.60
Testosterone (nmol/L)	1.31	0.30, 2.33	0.01

* GEE model incorporates changes at 8 and 12 weeks. A GEE model with an exchangeable correlation structure was used.

## Data Availability

The datasets used and/or analyzed during the current study are available from the corresponding author upon reasonable request.

## Supplementary Materials

The following are available online at https://www.mdpi.com/article/10.3390/nu13082550/s1, Supplemental Methods. Table S1: Safety profile of berberine in this trial and most recent systematic review and meta-analysis of clinical trials.
