# Supplementary material for: Effect of Berberine on Cardiovascular Disease Risk Factors: A Mechanistic Randomized Controlled Trial
Source: Nutrients. 2021 Jul 26;13(8):2550. doi: 10.3390/nu13082550 (PMC8401658; doi:10.3390/nu13082550)
Supplement: Supplementary file 1 [file nutrients-13-02550-s001.zip › nutrients-1277505-SI.pdf]

Supplementary Methods

Sample size calculation

Previous studies suggested an effect size of berberine on LDL-c is ~0.70 [1, 2], at a significance level of 0.05 and 80% power, a sample size of 33 in each arm is needed [3]. Besides, assuming the proportion of non-compliance during the trial is 5%, the sample size in each arm required for in each group is 37 (n=33/(1-0.05)^2=37) [4]. Considering the uncertainty, we recruited 37/0.9=42 in each group. As such, in the analysis 40 men in each arm is adequate to detect the effect size specified in previous studies [1,2].

Supplementary Table S1. Safety profile of berberine in this trial and most recent systematic review and meta-analysis of clinical trials.

Study	Study design	Summary
Current trial	Randomized controlled trial (RCT)	No serious adverse effect reported. Headache occurred in one participant in the berberine group. Headache, nausea and vomiting occurred in one participant in the placebo group. Diarrhoea occurred in one participant in the berberine group.
Yu, M et al (2020)	Most recent systematic review and meta-analysis	"The most commonly reported adverse effect was vomiting and rash, but were not serious. No deaths were reported in any of the included studies."

References

1. Gu Y, Zhang Y, Shi X, Li X, Hong J, Chen J, Gu W, Lu X, Xu G, Ning G: Effect of traditional Chinese medicine berberine on type 2 diabetes based on comprehensive metabonomics. *Talanta* 2010, 81(3):766-772.
2. Zhang Y, Li X, Zou D, Liu W, Yang J, Zhu N, Huo L, Wang M, Hong J, Wu P *et al*: Treatment of type 2 diabetes and dyslipidemia with the natural plant alkaloid berberine. *J Clin Endocrinol Metab* 2008, 93(7):2559-2565.
3. Zhong B: How to calculate sample size in randomized controlled trial? *J Thorac Dis* 2009, 1(1):51-54.
4. Wittes J: Sample size calculations for randomized controlled trials. *Epidemiol Rev* 2002, 24(1):39-53.
